# Supplementary material for: Trajectories of imitation skills in preschoolers with autism spectrum disorders
Source: J Neurodev Disord. 2022 Jan 5;14:2. doi: 10.1186/s11689-021-09412-y (PMC8903579; doi:10.1186/s11689-021-09412-y)
Supplement: Supplementary file 1 — Additional file 1. Supplementary Materials. [file 11689_2021_9412_MOESM1_ESM.docx]

# Supplementary materials

## Description of the Visuo-Motor Imitation scale

The Visuo-Motor Imitation scale is part of the Psychoeducational Profile, 3rd edition [(Schopler & al., 2005)](https://www.zotero.org/google-docs/?FAe4CZ), which is an assessment tool for children with developmental disabilities and, in particular, children with Autism Spectrum Disorders. It is validated for children from 2 to 7 years old. The Visuo-Motor Imitation scale includes 10 items of imitation described in the following list. These items are assessed on a 3-point scale (0 = absence of imitation, 1 = unclear, partial or prompted imitation, 2 = complete imitation of the skill).

1. Imitate the examiner looking through a kaleidoscope while turning the ring (two actions needed for a score of two)
2. Imitate the examiner pressing a desk bell twice
3. Imitate the examiner sticking his/her index into the *Play-Doh*, leaving a hole
4. Pretend to blow out the candles at the end of the "Happy Birthday" song
5. Show enjoyment while examiner sings the song "Happy Birthday" by singing or moving
6. Roll the *Play-Doh* after the demonstration by the examiner
7. Put the hand in the hand puppet (dog or cat) and animate head and arms
8. Imitate everyday actions with a hand puppet (eating, drinking etc.)
9. Demonstrate the use of four everyday objects (glass, spoon, pencil etc.)
10. Imitate three gross motor movements (raise the arm, touch the nose, raise the arm and touch the nose simultaneously)

## Statistical comparison between groups at baseline

As our baseline distributions didn't follow normal distributions, the statistical comparisons between groups at baseline were on medians which were presented in *Table S1*.

**Table S1.** *Statistical Comparison Between Children With ASD and Children with TD at Baseline in Terms of Demographic, Clinical, Cognitive Features*

|  | | ASD - *Mdn* *n* = 177 | TD - *Mdn* *n* = 43 | *p*-value* |
| --- | --- | --- | --- | --- |
| Sex | | 24 ♀ / 153 ♂ | 10 ♀/ 33 ♂ | 0.155^1^ |
| Age (years old) | | 2.82 | 2.76 | 0.429^2^ |
| ADOS Total symptom severity | | **8** | **1** | **<0.001**^2^ |
| PEP-3 | |  |  |  |
|  | CVP (raw score) | **27** | **45** | **<0.001**^2^ |
|  | EL (raw score) | **4** | **27** | **<0.001**^2^ |
|  | RL (raw score) | **10** | **32** | **<0.001**^2^ |
| VABS-II Adaptive Behavior Composite | | **77** | **110** | **<0.001**^2^ |

*Note.* ADOS = Autism Diagnosis Observation Schedule ; PEP-3 = PsychoEducational Profile, 3rd edition ; CVP = Cognition Verbal and Preverbal ; EL = Expressive language ; RL = Receptive language ; VABS-II = Vineland Adaptive Behavior Scales, 2nd edition.

* p value of *Fisher’s exact test^1^* and *Mann-Whitney tests^2^* on medians of differences between the ASD and TD groups. Significant results are shown in bold.

#### Imitation at baseline as a predictor of the autistic symptoms and developmental changes

To complement previous analyses and following others (Vivanti & al., 2013), we explored the extent to which early imitation skills might predict changes in the level of communication in these children one year later. This was performed using Spearman partial correlations between the score of imitation and the score of change in communication domains (the scores at time-point 2 subtracted from the ones at time-point 1). Similarly to the results included in the main manuscript, we observed that strong correlations between imitation at baselines and receptive and expressive language improvements a year after (see *Figure S1*).

We further examined how scores in receptive and expressive language changed over time in the 3 subgroups that we had defined as a function of imitation trajectories (see section “imitation trajectories within the ASD group”), using mixed-effects models from the longitudinal dataset. As shown in *Figure S2*, we observed that imitation skills have a prominent role on the subsequent development of language skills, by which the children who either had better imitation skills at baseline (ASD1), or fast improvement in imitation skills (ASD2) were the ones showing the best language outcomes.

**Figure S1.** Spearman partial correlations between imitation skills (raw scores of the Visuo-Motor Imitation scale of PEP-3) at baseline and a one-year gain in receptive (a) and expressive language (b) while controlling for the baseline levels in respective skills in ASD group (gain was defined as difference in age equivalent scores of the Mullen Scale of Early Learning a year later comparing to baseline).

**

**Figure S2.** Receptive (a) and Expressive (b) language skills over time within the ASD group (*n* = 102). The trajectories at the group level (solid lines black=ASD1, *n_ASD1_* = 46, red=ASD2, *n_ASD2_* = 27, blue=ASD3, *n_ASD3_* = 29) are obtained using mixed-effect models [(Mancini](https://www.zotero.org/google-docs/?lR3QYd) [&](https://www.zotero.org/google-docs/?QjtePU) [al., 2019; Mutlu](https://www.zotero.org/google-docs/?08xblg) [&](https://www.zotero.org/google-docs/?05CC1H) [al., 2013)](https://www.zotero.org/google-docs/?qfrQEd). The 95% confidence interval of estimated group-level trajectory is represented in colored bands. Repeated time-points of the same subjects are connected with dotted lines.

## Statistical comparison between ASD subgroups at baseline

In order to differentiate the subgroups ASD2 and ASD3 at baseline, we compared them according to the level of symptoms (ADOS Total symptom severity) and cognitive and language skills (PEP-3). In addition, we compared the number of children in each group that was included in an early and intensive Early Start Denver Model intervention program in Geneva [(Rogers & Dawson, 2010)](https://www.zotero.org/google-docs/?eOtQ5w). Finally, in order to test for potential differences between the two groups with regards to social orienting, we used eye-tacking. In this task adynamic social and geometric videos are presented side-by-side [(see Franchini & al., 2017, for more details)](https://www.zotero.org/google-docs/?kdft34). Thus, as a measure of social orienting, for each child we were able to calculate the percentage of time she/he spent on the videos containing social information compared to the videos with geometric forms .

**Table S2.** *Statistical Comparison Between Subgroups ASD2 and ASD3 at Baseline in Terms of Clinical, Cognitive, Therapy and Eye-tracking Features*

|  | | ADS2 - *Mean (SD)* *n* = 27 | ASD3 - *Mean (SD)* *n* = 29 | *p*-value |
| --- | --- | --- | --- | --- |
| ADOS Total symptom severity | | 8.46 (1.6) | 9.07 (1.2) | *p* = 0.179^1^ |
| PEP-3 | |  |  |  |
|  | VPC (raw scores) | 15.81 (8) | 13.61 (6.7) | *p* = 0.613^1^ |
|  | EL (raw scores) | 2.96 (4.7) | 1.71 (1.2) | *p* = 0.613^1^ |
|  | RL (raw scores) | 4.73 (6) | 3.71 (3) | *p* = 0.873^1^ |
| Number of subjects included in an early intervention (CIPA) | | 15 | 13 | *p* = 0.408^2^ |
| Social orientation (eye-tracking) | | 39.89 (19.3) | 45.68 (19) | *p* = 0.937^3^ |

*Note.* ADOS = Autism Diagnosis Observation Schedule ; PEP-3 = PsychoEducational Profile, 3rd edition ; CVP = Cognition Verbal and Preverbal ; EL = Expressive language ; RL = Receptive language.

* p value of *Mann-Whitney tests^1^* , *Chi-square test^2^* and *t-test^3^* of differences between the two subgroups.
